# Oncometabolite succinate promotes angiogenesis by upregulating VEGF expression through GPR91-mediated STAT3 and ERK activation

**DOI:** 10.18632/oncotarget.14485

**Published:** 2017-01-04

**Authors:** Xianmin Mu, Ting Zhao, Che Xu, Wei Shi, Biao Geng, Jiajia Shen, Chen Zhang, Jinshun Pan, Jing Yang, Shi Hu, Yuanfang Lv, Hao Wen, Qiang You

**Affiliations:** ^1^ Department of Biotherapy, Second Affiliated Hospital, Nanjing Medical University, Nanjing, Jiangsu 210011, China; ^2^ Department of Surgery, Second Affiliated Hospital, Nanjing Medical University, Nanjing, Jiangsu 210011, China; ^3^ Department of Immunology, Nanjing Medical University, Nanjing, Jiangsu 211166, China; ^4^ Department of Drug Screening and Evaluation, Chia Tai Tianqing Pharmaceutical Group Co., Ltd, Nanjing, Jiangsu 210023, China

**Keywords:** succinate, GPR91, gastric cancer, angiogenesis, ERK1/2

## Abstract

Altered cellular metabolism is now generally acknowledged as a hallmark of cancer cells, the resultant abnormal oncometabolites cause both metabolic and nonmetabolic dysregulation and potential transformation to malignancy. A subset of cancers have been found to be associated with mutations in succinate dehydrogenase genes which result in the accumulation of succinate. However, the function of succinate in tumorigenesis remains unclear. In the present study, we aim to investigate the role of oncometabolite succinate in tumor angiogenesis. Our data demonstrated the accumulation of markedly elevated succinate in gastric cancer tissues compared with that in paracancerous tissues. Moreover, succinate was able to increase the chemotactic motility, tube-like structure formation and proliferation of primary human umbilical vascular endothelial cells (pHUVECs) *in vitro*, as well as promoting the blood vessel formation in transgenic zebrafish. Our mechanistic studies reveal that succinate upregulates vascular endothelial growth factor (VEGF) expression by activation of signal transducer and activator of transcription 3 (STAT3) and extracellular regulated kinase (ERK)1/2 via its receptor GPR91 in a HIF-1α independent mechanism. Taken together, these data indicate an important role of the succinate-GPR91 axis in tumor angiogenesis, which may enable development of a novel therapeutic strategy that targets cancer metabolism.

## INTRODUCTION

Dysregulated metabolism is generally accepted as a characteristic hallmark of cancer cells [1] which exhibit altered metabolic phenotype when compared with nonmalignant cells [2]. Mutations in the metabolic genes like fumarate hydratase, succinate dehydrogenase, and isocitrate dehydrogenase lead to the accumulation of fumarate, succinate, and D–2-hydroxyglutarate, respectively [3], which cause both metabolic and nonmetabolic dysregulation and potential transformation to malignancy.

Angiogenesis is another biological hallmark of cancer [1]. The normal vasculature becomes quiescent following the morphogenesis. In contrast, the angiogenic switch during tumorigenesis is almost always activated and remains on, causing quiescent vasculature to sprout new vessels that help sustain expanding neoplastic growths continually [4]. Angiogenesis is a complicated multistep process which contains steps of the migration, proliferation, tube formation, invasion and finally capillary network formation of vascular endothelial cells [5], and is tightly regulated directly or indirectly by the dynamic balance between angiogenic stimulators and inhibitors.

As one of metabolic products in the tumor environment, lactate has been proved to promote cancer angiogenesis [6]. Another oncometabolite succinate was also found to accumulate in certain cancers which mainly caused by mutation of succinate dehydrogenase gene. However, its role in tumorigenesis remains unclear. As an intermediate of the tricarboxylic acid cycle, succinate is essential for adenosine triphosphate generation in mitochondria and also a crucial metabolite in several metabolic pathways. It is normally present in the circulation of blood at about 5μM concentration [7, 8], while under pathological conditions it is accumulated in extra-cellular spaces. High succinate levels (from 150μM to millimole) have been detected in the urine and plasma or the cerebral white matter of patients with metabolic diseases [9, 10]. Besides as energy metabolism, recent studies have demonstrated the new role of succinate as a cellular signaling molecule. For example, succinate accumulates in succinate dehydrogenase-deficient tumors like gastrointestinal stromal tumors (GIST), inhibits prolyl hydroxylase activity with subsequent HIF-1α stabilization and therefore drives cancer progression [11, 12]. Succinate also enhances interleukin-1β and VEGF production in a HIF-1α-depend pathway [9]. These significant findings indicate that succinate is capable of acting as an intracellular messenger which induces the alterations of gene expression in tumors by targeting HIF-1α. Meanwhile, succinate was demonstrated as an extracellular circulating signaling molecule in a para- and endocrine mode mediated by its specific plasmalemmal succinate receptor [13], which probably beyond HIF-1α stabilization.

G protein-coupled receptor-91 (GPR91), also known as succinate receptor 1 (SUCNR1) [10], is expressed in several highly vascularized tissues, including retina, heart, liver, kidney and white adipose tissue, and even on the surface of human monocyte-derived dendritic cells. GPR91 activation triggered by local extracellular succinate inhibits lipolysis in white adipose tissue, governs retinal angiogenesis, increases the release of renin in glomerular endothelium, enhances the secretion of pro-inflammatory cytokines by dendritic cells for activation of T helper cells, and also serves as a signaling regulator of hepatic fibrosis [13–20].

The comprehensive roles of succinate and its specific receptor in the development of tumor angiogenesis have not been investigated extensively. In the present study, we examined succinate levels in human gastric cancer tissues and demonstrated that succinate functioned as a biologically active molecule through its receptor GPR91, which bridges a gap between tumor-altered metabolism and angiogenesis.

## RESULTS

### Comparison of succinate levels in gastric cancer tissues/paracancerous tissues, cell culture supernatants from normal cells and gastric cancer cells

**Figure 1:**
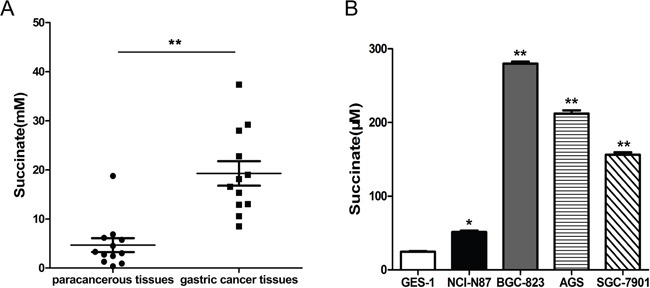
Succinate level is significantly higher in human gastric cancer tissues and cancer cell lines compared with adjacent tissues and normal cell line, respectively **A**. Succinate levels were measured in twelve pairs of human gastric adenocarcinoma and tumor-adjacent tissues, which were collected from stage II-IV gastric cancer patients. **p<0.01 compared with adjacent tissues. **B**. Succinate levels were determined in the culture supernatants of human gastric cancer cells and normal gastric epithelial cells. *p<0.05 and **p<0.01 versus the culture supernatants of GES-1 cells.

Succinate levels were measured in 12 pairs of human gastric adenocarcinoma and tumor-adjacent tissues, which were obtained from stage II-IV male patients with low/moderate-differentiated gastric adenocarcinoma over 45 years of age. As shown in Figure 1A, the levels of succinate in gastric cancer tissues were significantly higher than that in adjacent normal tissues. To detect the extracellular succinate levels of different cell lines, the cells were cultured in RPMI 1640 media (no phenol red) supplemented with 0.5% FBS for 24h and the supernatants were collected for measurement. Markedly elevated succinate levels in human gastric cancer cells (NCI-N87, BGC-823, AGS and SGC-7901) culture were observed compared to human normal gastric epithelial cell (GES-1) culture (Figure 1B). These findings implicate that gastric cancer cells exhibit altered metabolic patterns when compared with nonmalignant cells at least in terms of succinate levels.

### Succinate increases the chemotactic motility, capillary structure formation and proliferation of pHUVECs *in vitro*

**Figure 2:**
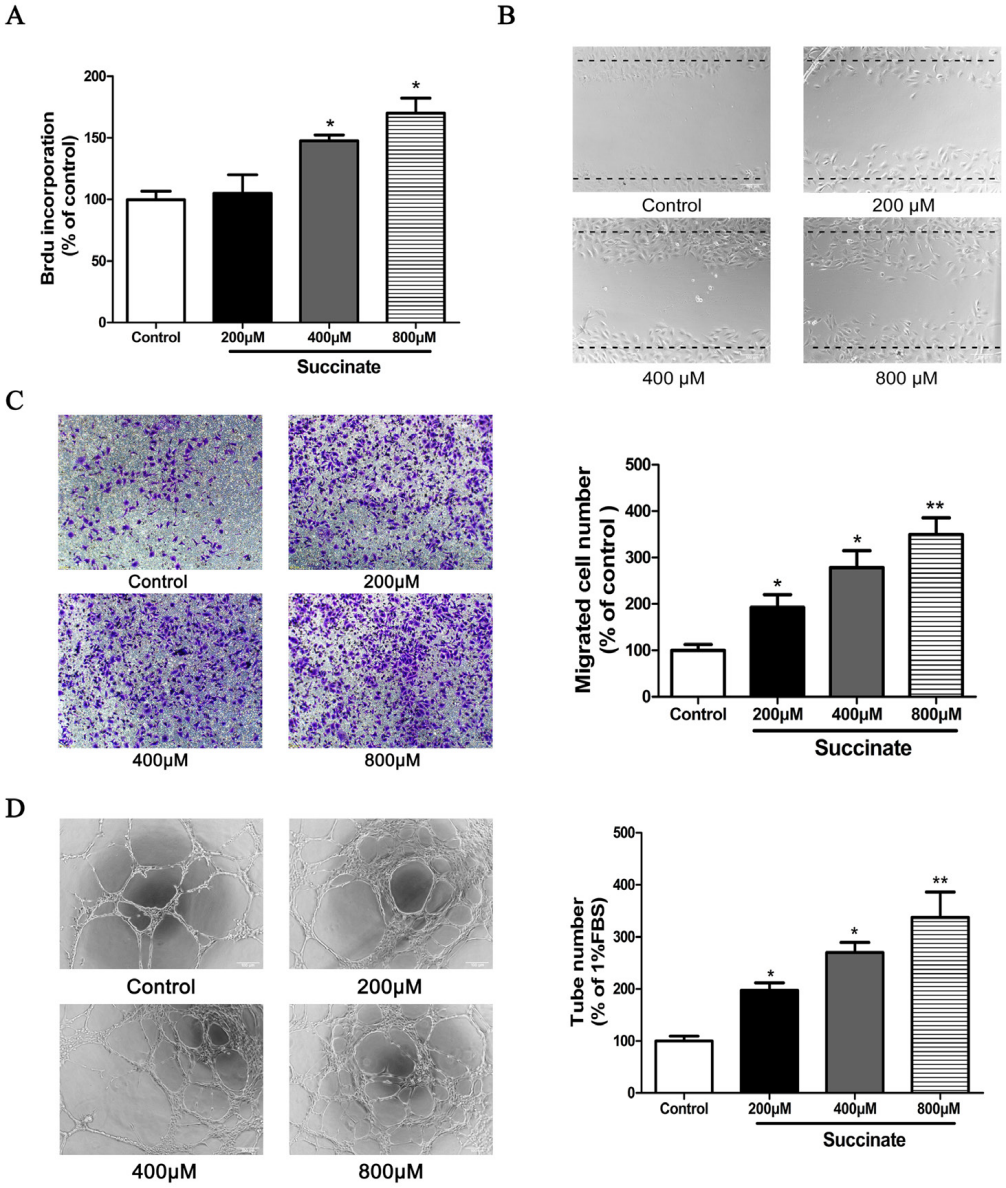
Succinate increases the proliferation, chemotactic motility and capillary structure formation of pHUVECs *in vitro* **A**. BrdU incorporation assay was used to determine the proliferation of pHUVECs in the presence of various concentration of succinate. **B**. Wound-healing migration assay was applied to observe the effect of succinate on pHUVECs migration. The confluent cells were scratched by pipette and cultured in the presence of various concentration of succinate (magnification, ×100). **C**. Effect of succinate on pHUVECs migration was also evaluated by transwell assay. pHUVECs were seeded in the upper transwell chamber. The bottom chamber was filled with various concentrations of succinate. After 8 h of co-culture, the migrated cells were quantified by manual counting (Magnification, ×100). **D**. Capillary-like formation of pHUVECs was observed in the presence of various concentration of succinate. pHUVECs (2.0 ×10^4^ cells/well) were placed in 96-well plates coated with growth factor reduced matrigel. Six hours later, tubular structures were photographed (Magnification, ×100). Three independent experiments (A, B, C and D) data are presented as mean ±SD. *p<0.05 and **p<0.01 versus PBS control.

The effect of succinate on pHUVEC proliferation was assessed by BrdU incorporation assay. As a result, treatment with 400μM or 800μM of succinate significantly increased the viability of primary pHUVEC when compared to control (Figure 2A). Since the migration of endothelial cells is a crucial step in angiogenesis and cancer progression [21], the effects of succinate on chemotactic motility of pHUVECs were evaluated by transwell and wound-healing migration assays. As shown in Figure 2B and 2C, succinate significantly increased pHUVEC migratory straight distance when compared to PBS control in a concentration-dependent manner. Since the differentiation of endothelial cells and the formation of tube-like structures are essential for angiogenesis, the ability of succinate to promote tube formation was further investigated in a three-dimensional model of endothelial cell capillary tube formation in Matrigel. Stimulation with 10 ng/mL VEGF promoted pHUVECs differentiating and forming robust tubular-like structures (data not shown). Similarly, succinate enhanced the formation of a tube-like network as well after 6 h of culture as shown in Figure 2D. Quantitative measurements confirmed that succinate triggered a significant increase in total tubes.

### Succinate promoted the vessel formation in zebrafish embryo

**Figure 3:**
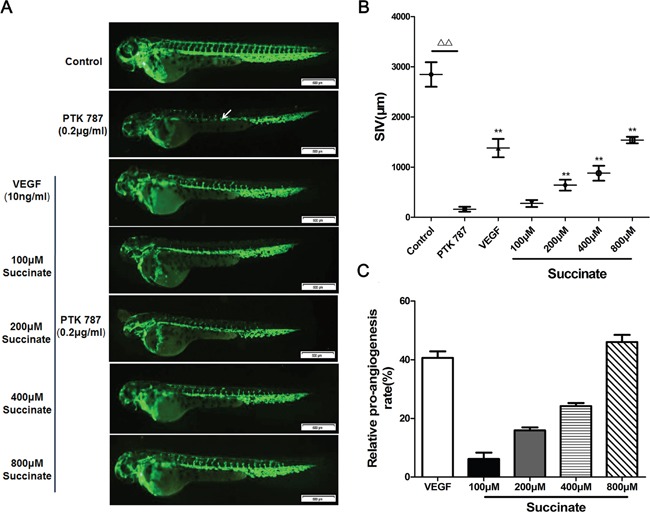
Succinate promotes vessel formation in the zebrafish embryo **A**. Effect of succinate on angiogenesis was evaluated in zebrafish embryos. Transgenic (flk1: GFP) zebrafish embryos were picked out and distributed into a 24-well microplate (9 embryos/well), and pre-treated with 0.2 μg/mL PTK787 (VEGFR inhibitor, Sigma). Then, various concentration of succinate (0, 100, 200, 400, 800 μM) or 10 ng/mL of VEGF_165_ were added to the culture for twenty-four hours. Representative photographs are listed to show the subintestinal vessels (SIVs) indicated by white arrow. **B, C**. Quantitative analysis of the overall length of zebrafish SIVs are shown. Data are presented as mean ±SD.*p<0.05 and **p<0.01 versus PBS control.

To study the effect of succinate on angiogenesis *in vivo*, transgenic (flk1: GFP) zebrafish embryos, which express GFP in all vascular endothelial cells, were used. The development of zebrafish SIVs was visualized using fluorescence stereomicroscope. SIVs length was measured with Image Pro Plus. The representative photos and quantitative analysis of zebrafish SIVs were shown in Figure 3. The small molecule kinase inhibitor PTK787 was used in pro-angiogenesis *in vivo* model, which inhibits human VEGFRs and therefore effectively blocks angiogenesis [22]. As shown in Figure 3A, treatment with 0.2 μg/mL PTK787 prevented the formation of blood vessels. As anticipated, the simultaneous treatment of VEGF_165_ significantly restored angiogenesis. In line with the observations described above, the length of SIVs was significantly increased in zebrafish embryos treated with succinate at the concentration above 100μM when compared to PBS control, which indicates a pro-angiogenic effect of succinate on zebrafish embryo in a dose-dependent manner.

### Succinate activates STAT3 and ERK signaling through GPR91

**Figure 4:**
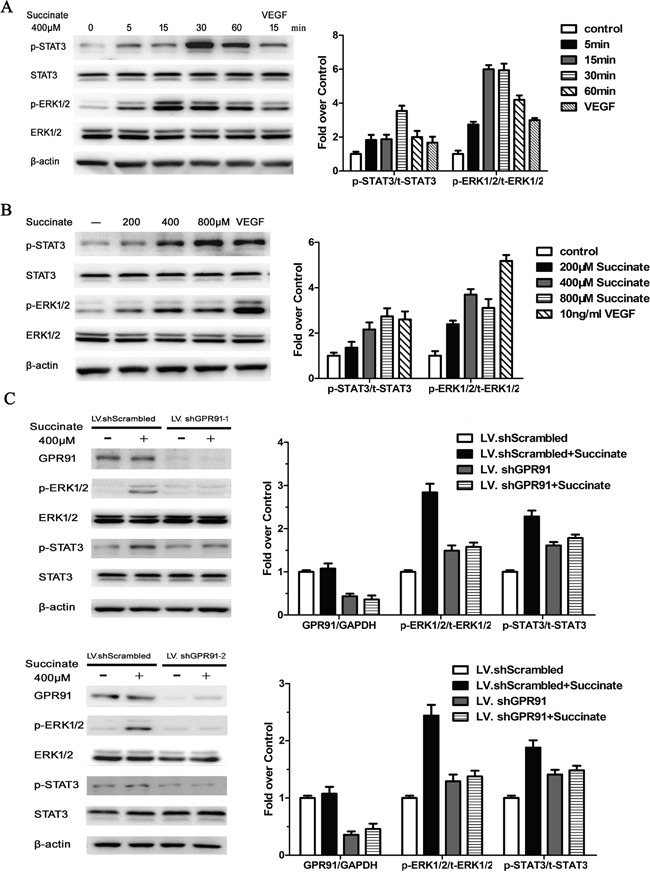
Succinate activates ERK1/2 and STAT3 signaling through GPR91 in pHUVECs **A**. STAT3 and ERK1/2 activation were measured by Western blotting in pHUVECs treated with 400μM succinate for different periods of time. **B**. STAT3 and ERK1/2 activation were determined in pHUVECs cultured in the presence of various concentrations of succinate or 10 ng/mL of VEGF_165_ for 15 min. **C**. STAT3 and ERK1/2 activation were evaluated in pHUVECs transduced with LV.shScrambled or LV. shGPR91-1, or LV. shGPR91-2 in the presence of succinate for 15 min. The expression of total STAT3 and Erk1/2 protein were also detected. β-actin was used as loading control. Data were represented as mean ± SD; n = 3. *P<0.05 versus PBS control.

In order to investigate the pro-angiogenic mechanism of succinate, we first determined the timing and dosage of succinate treatment on STAT3 and ERK activation. pHUVECs were cultured in the presence of 400 μM succinate and measured ERK1/2 and STAT3 expression at different time point (5, 15, 30 and 60 min). As shown in Figure 4A, the peak of pERK1/2 expression level is at 15 min, while the peak of pSTAT3 expression is at 30 min. The data suggest ERK activation by succinate is earlier than STAT3. A dose-dependent trend of the activation was further observed when pHUVECs were treated with various concentrations of succinate (Figure 4B). To verify that succinate activated ERK1/2 and STAT3 signaling through the receptor GPR91, we knocked down its expression with two kinds of lentiviruses containing the different shRNA sequences (LV. shGPR91-1 and 2), which were also adopted to exclude the off-target effect. Both LV. shGPR91 (transfection rate of ~95%) diminished GPR91 mRNA approximately 85-90% and the protein expression about 65%, whereas LV.shScrambled (control) was ineffective to reduce GPR91 expression (Figure 4C). Using this knockdown technique, we observed that the increase in the phosphorylation levels of ERK1/2 and STAT3 was significantly blocked by both LV. shGPR91 (P<0.01) as shown in Figure 4C. These results indicate that GPR91 mediates the activation of ERK1/2 and STAT3 signaling by succinate.

### Succinate promotes angiogenesis by activating ERK1/2 signaling via GPR91 in pHUVECs

**Figure 5:**
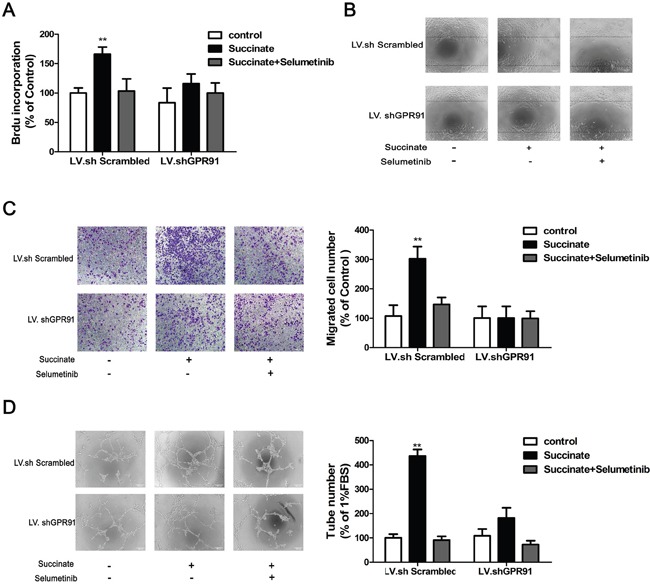
Succinate promotes angiogenesis by activating ERK1/2 signaling through GPR91 pHUVECs, transduced with LV.shGPR91 or LV.shScrambled, were pre-treated with 10μM ERK1/2 inhibitor selumetinib and then cultured in the presence of succinate to evaluate its pro-angiogenic effect. **A**. BrdU incorporation assay was used to determine the effect of succinate on the proliferation of pHUVECs. **B**. The wound-healing assay was applied to observe the effect of succinate on pHUVECs migration (magnification, ×100). **C**. Transwell assay was also adopted to determine the effect of succinate on pHUVECs migration (Magnification, ×100). **D**. Capillary-like formation of pHUVECs was observed in the presence of succinate (Magnification, ×100). The data from three independent experiments are presented as mean±SD. *p<0.05 and **p<0.01 versus LV.shScrambled group.

A series of assays including BrdU incorporation, wound-healing migration, transwell and capillary-like formation were applied to evaluate the pro-angiogenic effect of succinate. pHUVECs transduced with LV.shGPR91 or LV.shScrambled were pre-treated with 10μM ERK1/2 inhibitor selumetinib to assess the activation of succinate on ERK1/2 through GPR91. As shown in Figure 5A–5D, treatment with 400μM of succinate significantly increased the viability, chemotactic motility and capillary structure formation ability of pHUVEC compared to the control. However, addition with selumetinib diminished these effects. Interestingly, the pro-angiogenic effect of succinate on pHUVECs was significantly blocked in the cells transduced with LV. shGPR91 (p<0.01, Figure 5A–5D). These data suggest that the activation of ERK1/2 signaling mediated by GPR91 is important for succinate to promote angiogenesis in pHUVECs.

### VEGF upregulation induced by succinate depends on the activation of STAT3 and ERK1/2 signaling in pHUVECs

**Figure 6:**
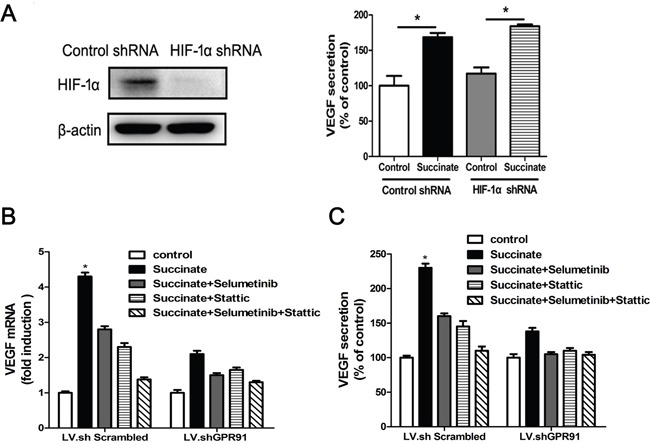
Succinate enhances VEGF expression by activating ERK1/2 and STAT3 signaling through GPR91 **A**. Succinate increases VEGF expression independent of HIF-1α gene. The expression of HIF-1α protein in LV.shScrambled-transduced or LV.shHIF-1α-transduced AGS cells was determined by Western blotting (left). The cell culture supernatant was collected from the transduced cells in the presence of 400 μM succinate for 24h and measured VEGF level (right). VEGF mRNA levels **B**. or protein concentration in the supernatant **C**. were determined by RT-qPCR or ELISA in pHUVECs cultured in the presence of succinate for 12h, which were pre-treated with 10μM Selumetinib (ERK1/2 inhibitor) or/and 10μM Stattic (STAT3 inhibitor) for ten minutes. Each column represents the mean±SD (n=3). *P<0.05 versus PBS control.

Next, we sought to investigate the mechanism of pro-angiogenic function of succinate on pHUVECs. VEGF is a well-known angiogenic mediator that can promote endothelial cells growth. As shown in Figure 6A, 6B and 6C, succinate treatment significantly increased VEGF mRNA and protein levels in pHUVECs compared with PBS control group. Since VEGF is the target gene of HIF-1α transcription factor, we knocked down HIF-1α gene and determined whether HIF-1α played a functional role in the effect of succinate. Interestingly, depletion of HIF-1α didn't affect the upregulation of VEGF induced by succinate as shown in Figure 6A. Instead, ERK inhibitor Selumetinib or STAT3 inhibitor Stattic alone was able to decrease VEGF expression induced by succinate, and the combinations further reduced to the control level (Figure 6B and 6C). Then, we investigated the role of GPR91 in the regulation of VEGF secretion by succinate. GPR91 knockdown reduced VEGF mRNA by approximately 55% (Figure 6B) and significantly decreased VEGF protein expression by approximately 34% (Figure 6C). These data indicated that effect of succinate on VEGF upregulation was through modulating STAT3 and ERK1/2 signaling via GPR91 in pHUVECs.

## DISCUSSION

Altered cellular metabolism is now known as a hallmark of cancer cells. The involved metabolic pathways include aerobic glycolysis, glutamine catabolism, anaplerosis, de novo synthesis of fatty acids and lipids [23]. The resultant abnormally accumulated metabolites cause both metabolic and nonmetabolic dysregulation and potential transformation to malignancy, and herein termed oncometabolites including fumarate, succinate, and D-2-hydroxyglutarate [24]. An increasing body of evidence suggests that the abnormal accumulation of these metabolites can re-edit tumor microenvironment, therefore drive cancer progression.

Succinate, a crucial intermediate metabolite in several metabolic pathways, is accumulated in extracellular spaces under pathological condition, for instance, hyperglycemic or hypoxia. High concentrations of succinate (from 150μM to millimole) have been detected in the urine and plasma or the cerebral white matter of patients with metabolic diseases [9, 10]. Dysfunction of the succinate dehydrogenase, a heterotetrameric enzyme complex participating in both the citric acid cycle and electron transport chain, leads to succinate accumulation in human tumors such as papillary thyroid cancers, thyroid C-cells hyperplasia, pancreatic neuroendocrine tumors, paragangliomas, chronic lymphocytic leukemia, renal carcinomas and pituitary adenomas [7, 25]. However, there is no evidence to show whether oncometabolite succinate elevation is present in gastric cancer. Using tissue samples from patients with stage II-IV gastric cancer we demonstrated that succinate levels were increased in human gastric adenocarcinoma compared with tumor-adjacent tissues (Figure 1A). In addition, our data reveal that human gastric cancer cells (NCI-N87, BGC-823, AGS and SGC-7901) produce more succinate than normal epithelial GES-1 cells (Figure 1B).

Angiogenesis inducing is another hallmark of cancer [1, 4]. It has been shown that succinate accumulates in the hypoxic retina of rodents as a potent mediator of vessel growth in the settings of both normal retinal development and proliferative ischemic retinopathy [15]. Therefore, we focus on investigating the function of succinate in tumor angiogenesis. Herein, we examined pro-angiogenic effects of succinate *in vitro* and *in vivo*. As shown in Figure 2 and 3, succinate was able to increase chemotactic motility, tube-like structure formation and proliferation of primary human umbilical vascular endothelial cells (pHUVECs), as well as promoting blood vessel formation in transgenic zebrafish.

The well-known prototype of angiogenesis inducers is VEGF, which is a well-documented angiogenic mediator that can promote endothelial cells growth, maturation and survival [26]. In the present study, we demonstrate that succinate mediates HIF-independent upregulation of VEGF and promote tumor angiogenesis via plasmalemmal GPR91 at pH 7.4. Succinate has been identified as an inhibitor of prolyl 4-hydroxylases (P4Hs) *in vitro* to upregulate HIF-1α expression, which has Ki values of 350-460 μM [11]. It doesn't penetrate cell membrane easily and has limited uptake into cells, especially in PH7.4 [27, 28]. Therefore, we administered certain amount of succinate which didn't induce HIF-1α stabilization. Interestingly, VEGF expression induced by succinate is through GPR91 receptor in HIF-1α-independent manner (Figure 6A). It is noteworthy that the effective concentration of succinate for a half-maximal (EC50) response on GPR91 is 28-56 μM [10], higher than the 10μM succinate used in aortic rings assay which resulted in minimal sprouting [15], indicating that GPR91 would be activated at higher concentrations of succinate in pHUVECs. We find that 200μM succinate already can stimulate VEGF production and promote tumor angiogenesis beyond HIF-1α.

MEK-ERK signaling pathway plays a central role in tumor angiogenesis [29]. Its activation promotes sprouting and increases vessel length in tumors [30]. Succinate has been shown to activate ERK1/2 through Gq and Gi [31]. In the present study, we demonstrated that succinate promoted angiogenesis by ERK1/2 signaling via GPR91 in pHUVECs. Interestingly, succinate also activated STAT3 tyrosine705 phosphorylation (Figure 3). STAT3 is known as a direct transcriptional activator of VEGF gene by its phosphorylation at tyrosine705, which is essential for its dimer formation, nuclear translocation, and DNA binding activity [32]. Transactivation of the STAT3 C-terminal domain depends on its tyrosine705 and serine 727. ERK1/2 specifically phosphorylates STAT3 at serine 727, which negatively modulates STAT3 tyrosine phosphorylation [33]. We presume that ERK1/2 activation would reduce STAT3 tyrosine705 phosphorylation at a later time in pHUVECs since the phosphorylation of STAT3 tyrosine705 was not be detected at 24h after succinate stimulation, instead ERK1/2 signaling activity was still upregulated (data not shown). These data suggest that STAT3 activation participates in the early stage of succinate induced pro-angiogenesis, whereas sustained activation of ERK1/2 via GPR91 mainly accounts for the angiogenic function of succinate in the tumor microenvironment.

Angiogenesis is required for invasive tumor growth and metastasis, and plays an essential role in the control of cancer progression. Our data demonstrate the pro-angiogenic role of oncometabolite succinate, which can upregulate VEGF expression by activating ERK1/2 and STAT3 signaling via GPR91 receptor. These findings have potential clinical implications that targeting succinate-GPR91 axis may be applied as a novel therapeutic strategy to inhibit tumor angiogenesis.

## MATERIALS AND METHODS

### Human tissues

Clinical samples from 12 patients with histological proven low/ moderate-differentiated gastric adenocarcinoma were obtained at the Second Affiliated Hospital of Nanjing Medical University between May 2014 and November 2014. Both tumor tissues and non-cancerous tissues were confirmed histologically. This study was approved by the ethics committee of the Second Affiliated Hospital of Nanjing Medical University (2016 KY-043).

### Reagents and antibodies

Rabbit anti-human p-ERK1/2 monoclonal antibody, rabbit anti-human p-STAT3 (705) monoclonal antibody, rabbit anti-human STAT3 monoclonal antibody, rabbit anti-human β-actin monoclonal antibody and rabbit anti-human ERK1/2 monoclonal antibody were purchased from Cell Signaling Technology (Boston, MA, USA). Rabbit anti-human GPR91 polyclonal antibody were purchased from Novus Biologicals LLC (Littleton, CO, USA). Secondary antibodies were obtained from Invitrogen (Carlsbad, CA, USA). Growth factor-reduced Matrigel was purchased from BD Biosciences (Bedford, MA, USA), and recombinant human VEGF_165_ was obtained from R&D Systems (Minneapolis, MN, USA). Endothelial cell medium (ECM, 1001) was purchased from ScienCell (Carlsbad, CA, USA). Succinate was obtained from Sigma. Selumetinib and Stattic were from Selleck Chemicals (Houston, TX, USA).

### Cell lines and cell culture

Primary human umbilical vascular endothelial cells (pHUVECs) were isolated from human umbilical cord veins by collagenase (Sigma) digestion as described previously [34]. Briefly, pHUVECs were maintained in endothelial cell medium at 37°C under a humidified 95%:5% (v/v) mixture of air and CO_2_. Ten μM of Selumetinib or Stattic was applied to inhibit ERK1/2 or STAT3 activation.

Human gastric mucosal epithelial cell line GES-1 and human gastric cancer cell lines AGS (low-differentiated human gastric adenocarcinoma), NCI-N87 (well-differentiated human carcinoma), BGC-823 (low-differentiated human gastric adenocarcinoma) and SGC-7901 (moderate-differentiated human gastric adenocarcinoma) were cultured in RPMI 1640 medium supplemented with 10% FBS, 100 units/mL penicillin, and 100 μg/mL streptomycin. NCI-N87 and AGS cells were from the American Type Culture Collection. GES-1, BGC-823 and SGC-7901 cells were obtained from the Shanghai Institutes for Biological Sciences, Chinese Academy of Sciences, China.

### Lentiviruses and endothelial cells infection

Lentiviruses carrying shRNA sequence targeting human GPR91 or human HIF-1α were obtained from GeneChem (Shanghai, China). The viruses were used to infect cells in the presence of Polybrene. Forty-eight hours later, pHUVECs were cultured in medium containing puromycin for cells screening. The clones stably knocking down GPR91 were identified and verified by Western blotting and qPCR. The Short-hairpin RNA sequences are listed: shGPR91-1: GCCTCTCAACTTGGTCATCATctcgagATGATGACCAAGTTGAGAGGC; shGPR91-2: CCTTAACTCATAGACATCAATctcgagATTGATGTCTATGAGTTAAGG; shHIF-1α-1: CAGCTGACCAGTTATGATTGTctcgagACAATCATAACTGGTCAGCTG; shHIF-1α-2: AACTAACTGGACACAGTGTGTctcgagACACACTGTGTCCAGTTAGTT. The non-targeting control sequence is 5′-TTCTCCGAACGTGTCACGT-3′.

### Succinate level measurement

Succinate level was determined by Succinate Colorimetric Assay Kit (Sigma-Aldrich Inc., St Louis, MO, USA) according to the manufacturer's instructions. Briefly, tissues (50 mg) were rapidly homogenized on ice in 500 μL of ice-cold Succinate Assay Buffer and centrifuged at 10,000×g for 5 minutes to remove insoluble material. For cell cultures, the supernatants of cells (1×10^6^ cells) were collected and diluted 1:1 with 0.5 M Tris-HCl (pH 8.0). Then, the samples were added into a 96-well plate in duplicate wells and mixed with the appropriate Reaction Mix. The resultant mixtures were further incubated at 37°C for 30 minutes. The succinate concentration was determined by the standard curve using spectroscopy at 450 nm wavelength, and each measurement was performed in triplicate.

### Cell proliferation assay by bromodeoxyuridine (BrdU) incorporation

Cell proliferation was evaluated by a BrdU assay kit (Roche Applied Science, Mannheim, Germany) according to the manufacturer's instructions. Briefly, pHUVECs (5×10^3^ cells/well) were seeded in 96-well plates overnight. Then, the cells were starved in serum-free ECM for 6 h and further cultured in 100 μL of serum-free ECM containing various concentrations of succinate for 24 h. Ten μL of a BrdU-labeling solution was added to each well for 4 h and the wells were dried, fixed, and measured the optical density at a wavelength of 450 nm. These assays were repeated in triplicate.

### Wound-healing migration assay

pHUVECs were seeded into 6-well plates pre-coated with 0.1% gelatin (Sigma) to grow to full confluence and subsequently starved in serum-free ECM for 6 h to synchronization. The cells were wounded with pipette tips and cultured in serum-free ECM with or without various concentrations of succinate. Images were captured (Olympus; magnification, ×100) after 12 h of incubation at 37°C in a 95%:5% (v/v) mixture of air and CO_2_.

### Transwell migration assay

The chemotactic motility of pHUVECs was determined using a Transwell migration assay as described previously [34]. Briefly, the upper chambers were seeded with pHUVECs (1.8×10^4^ cells) suspended in 200 μL of serum-free ECGM; the bottom chambers were filled with 600 μL of serum-free ECGM supplemented with various concentrations of succinate. After 8 h of culture, the non-migrated cells were removed, and the migrated cells were fixed and stained with 1% crystal violet. Images were photographed (Olympus; magnification, ×100) and the migrated cells were counted manually.

### Capillary-like tube formation assay

Tube formation was assessed as previously described [34]. Growth factor-reduced Matrigel was pipetted into pre-chilled 96-well plates (60 μL per well) and polymerized for 1 hour at 37°C. pHUVECs (2.0×10^4^ cells/well) were first incubated in serum-free ECM for 6 h, subsequently seeded onto the Matrigel layer in the presence of various concentrations of succinate for 6h in 96-well plates. The tubular structures of the endothelial cells were recorded using an inverted microscope (Olympus; magnification, ×100). The number of meshes per field was calculated randomly from five fields.

### Zebrafish embryos angiogenesis assay

Transgenic (flk1: GFP) zebrafish embryos were generated by natural pair-wise mating, collected and maintained in 28.5°C incubator as previously described [35]. Healthy, limpid, and fluorescent embryos were picked out at 24 hours post-fertilization (hpf) and distributed into a 24-well microplate (9 embryos/well) containing Holt Buffer. A final concentration of 0.2 μg/mL PTK787 (VEGFR tyrosine kinase inhibitor, Sigma) was added to each well at 24 hours post-fertilization (hpf) and washed out at 30 hpf. Meanwhile, various concentration of succinate (100, 200, 400 and 800μM) or 10 ng/mL of VEGF_165_ were added to the culture media. PBS served as the control. Embryos were maintained in a 28.5°C incubator for an additional 24h, placed onto a glass slide, and photographed using fluorescence stereomicroscope. The overall length of the subintestinal vessels (SIVs) were quantified using Image J software. Pro-angiogenic effects were defined as the increase of SIVs length. The experimental protocol was established according to institutional ethical guidelines for animal experiments and approved by the Ethics Committee of the Second Affiliated Hospital of Nanjing Medical University.

### Quantitative real-time PCR for mRNA expression analyses

Total RNA was extracted from pHUVECs treated with succinate or inhibitors for 12h. For first-strand cDNA synthesis, 500 ng of total RNA was retrotranscribed using Prime Script RT reagent Kit (Thermo Fisher Scientific, Waltham, MA, USA). The resultant cDNA was subsequently amplified with the Maxima SYBR-Green/Rox qPCR Master Mix 2X kit (Thermo Fisher Scientific, Waltham, MA, USA) using the StepOnePlus Real-Time PCR System (Thermo Fisher Scientific). Changes in the expression of VEGF gene was determined relative to the mean critical threshold (CT) values of GAPDH gene. Human VEGF gene primers are 5′-AGGGCAGAATCATCACGAAGT- 3′ and 5′-AGGGTCTCGATTGGATGGCA-3′. Human GAPDH gene primers are 5′- TTGCCATCAATGACCCCTTCA- 3′ and 5′- CGCCCCACTTGATTTTGGA- 3′.

### Western immunoblot analysis

To study the molecular mechanism of succinate-inducing angiogenesis, pHUVECs were first starved in serum-free ECM for 6 h and then treated with or without various concentration of succinate for 15 min (for GPR91 activation) or 400μM succinate at different timepoints. After stimulation, the cells were harvested and lysed followed by centrifugation. The total protein concentration was determined by Bicinchoninic acid assay. An equal amount of protein from each lysate (30-40μg) was electrophoresed on 8-12% SDS polyacrylamide gels and probed with specific primary antibodies followed by horseradish peroxidase-conjugated goat anti-rabbit antibody.

### Enzyme-linked immunosorbent assay (ELISA)

The cell culture supernatant was collected from non-transduced, LV.shScrambled-transduced or LV.shGPR91-transduced cells treated with different concentration of succinate. Then, VEGF level was measured by VEGF enzyme-linked immunoassay kits (R&D Systems, Minneapolis, MN, USA) according to the manufacturer's instructions. Each sample was tested in triplicate.

### Statistical analysis

All data reported herein represent means ± SD from at least three independent experiments. Statistical comparisons between the treated groups and the control group were performed by one-way analysis of variance (ANOVA) followed by Dunnet's test, the difference between two groups was analyzed by Student's t-test. A P value of <0.05 was considered statistically significant.

## Abbreviations

pHUVECs: primary human umbilical vascular endothelial cells
HIF: hypoxia-inducible factor
ERK: extracellular regulated kinase
STAT3: Signal transducer and activator of transcription 3
GPR91: G protein-coupled receptor-91
VEGF: vascular endothelial growth factor
MAPK: mitogen-activated protein kinases
ECM: endothelial cell medium
BrdU: bromodeoxyuridine.
